# Bayesian Parameter Inference and Model Selection by Population Annealing in Systems Biology

**DOI:** 10.1371/journal.pone.0104057

**Published:** 2014-08-04

**Authors:** Yohei Murakami

**Affiliations:** Department of Biophysics, Division of Biology, Graduate School of Science, Kyoto University, Kyoto, Japan; Swiss Federal Institute of Technology (ETH Zurich), Switzerland

## Abstract

Parameter inference and model selection are very important for mathematical modeling in systems biology. Bayesian statistics can be used to conduct both parameter inference and model selection. Especially, the framework named approximate Bayesian computation is often used for parameter inference and model selection in systems biology. However, Monte Carlo methods needs to be used to compute Bayesian posterior distributions. In addition, the posterior distributions of parameters are sometimes almost uniform or very similar to their prior distributions. In such cases, it is difficult to choose one specific value of parameter with high credibility as the representative value of the distribution. To overcome the problems, we introduced one of the population Monte Carlo algorithms, population annealing. Although population annealing is usually used in statistical mechanics, we showed that population annealing can be used to compute Bayesian posterior distributions in the approximate Bayesian computation framework. To deal with un-identifiability of the representative values of parameters, we proposed to run the simulations with the parameter ensemble sampled from the posterior distribution, named “posterior parameter ensemble”. We showed that population annealing is an efficient and convenient algorithm to generate posterior parameter ensemble. We also showed that the simulations with the posterior parameter ensemble can, not only reproduce the data used for parameter inference, but also capture and predict the data which was not used for parameter inference. Lastly, we introduced the marginal likelihood in the approximate Bayesian computation framework for Bayesian model selection. We showed that population annealing enables us to compute the marginal likelihood in the approximate Bayesian computation framework and conduct model selection depending on the Bayes factor.

## Introduction

Mathematical modeling is a very useful and powerful approach in systems biology [1], [2]. Mathematical models used in systems biology are often represented by ordinary or partial differential equations. These differential equations contain a number of parameters which represent the rates of biochemical reactions or amounts of components (proteins, mRNAs etc). An appropriate mathematical model together with appropriate values of parameters enables us to explain, understand and predict the target biological phenomena in system level. However, we may have a number of competing and potential mathematical models to explain the observed experimental data. In addition, concrete values of parameters in mathematical models are often not well-known in previous experimental literatures. In such cases, we need to conduct model selection and parameter inference by some sort of systematic procedures.

For parameter inference, we can use likelihood based approach as a statistical approach [3]. In addition, many optimization algorithms have already been developed and used to conduct parameter inference in systems biology [4]–[8]. For model selection, AIC [9] has already used to compare a number of mathematical models in systems biology [10]–[12].

Alternative to those methods and approaches, Bayesian statistics enables us to conduct both model selection [11], [13]–[19] and parameter inference [13], [15], [16], [20], [21] under the coherent philosophy. For Bayesian model selection and parameter inference in systems biology, the framework named approximate Bayesian computation (ABC) or likelihood-free computation is often used [14], [15], [18], [22]–[27]. ABC is very useful when likelihood functions are analytically or computationally intractable, or it takes much cost for evaluation [14], [15], [23], [24]. Instead of evaluating the likelihood function, ABC compares the observed data and the simulated data, and gives us the approximated posterior distribution [14], [15].

However, to compute Bayesian posterior distributions, Monte Carlo methods often need to be used. As Bayesian parameter inference and model selection algorithms in the ABC framework, Markov chain Monte Carlo (MCMC) [15], [22], [24] and sequential Monte Carlo (SMC) [15], [18], [23] have already used. It is known that the efficiency of ABC-MCMC algorithm reduces when the sampler is trapped in a low probability area [23]. To overcome the problem, ABC-SMC algorithm was developed [15], [18], [23]. However, even though the algorithms work successfully, the posterior distributions of parameters may be almost uniform or very similar to their prior distributions [13]. In such cases, it is difficult to choose one specific value of parameter with high credibility as the representative value of the posterior distribution.

To overcome the problems, we introduced one of the Monte Carlo algorithms, population annealing [28], [29]. Population annealing is a so-called population Monte Carlo algorithm [30] as same as SMC. Although population annealing is usually used in statistical mechanics [28], [31], we showed that population annealing can be used to compute Bayesian posterior distributions for parameter inference and model selection in the ABC framework. To deal with un-identifiability of the representative values of parameters, instead of choosing one specific value of parameter in the posterior distribution, we ran the simulations with the parameter ensemble sampled from the posterior distribution, named “posterior parameter ensemble”. We propose this approach is valid if our purpose of parameter inference is to reproduce or predict the system dynamics, not to estimate the correct values of parameters. We showed that population annealing is an efficient and convenient algorithm to generate the posterior parameter ensemble. In addition, we showed that the simulations with the posterior parameter ensemble can, not only reproduce the data used for parameter inference, but also capture and predict the data which was not used for parameter inference. Lastly, we introduced the marginal likelihood in the ABC framework for Bayesian model selection. We showed that population annealing enables us to compute the marginal likelihood in the ABC framework and to conduct model selection depending on the Bayes factor. The validity of our propositions was firstly judged by applying our method to the feed-forward loop network motif models [32]–[36], secondly to the insulin dependent AKT pathway model [47], [48].

## Methods

### Bayesian parameter inference

For Bayesian parameter inference, under the given likelihood function *f*(*D_obs_*|*θ,M*) and the prior distribution of parameters *π*(*θ|M*), we try to obtain the posterior distribution of parameters *π*(*θ*|*D_obs_*,*M*), represented as
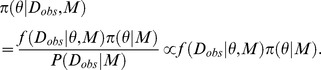

*M* is a model and fixed in parameter inference. *θ* is a set of parameters (i.e. multidimensional vector) in the model *M*. *D_obs_* is an observed experimental data. In the equation, *P*(*D_obs_|M*) is a normalization constant, and also called as a marginal likelihood.

In the ABC framework [14], [24], [26], instead of the true posterior distribution, we try to obtain the augmented posterior distribution *π_ABC_*(*θ,D_sim_|D_obs_*,*M*) [24]. The augmented posterior distribution can be represented as
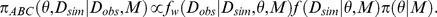

*D_sim_* is the simulated data, also called as the auxiliary parameter [24]. *f_w_*(*D_obs_|D_sim_*,*θ,M*) is the weighting function [24]. The weighting function is set to be large value when the observed data and the simulated data are “close”, small value when they are “distant”, and constant when they are “equal” (*D_obs_ = D_sim_*) [24]. As the weighting function, so-called indicator function is often used [14], [22]–[24], [26]. Indicator function is represented as
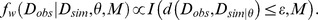

*d*(*D_obs_,D_sim|θ_*) is a distance measure between the observed data and the simulated data. *ε*≧0 is a tolerance. The indicator function equals to 1 if the observed data and the simulated data are close (≦*ε*) and 0 if not (>*ε*). If *ε* is small enough, the augmented posterior distribution is a good approximation of the true posterior distribution [14], [24]. Thus, we can use the augmented posterior distribution alternative to the true posterior distribution for parameter inference.

### Bayesian model selection

The marginal likelihood *P*(*D_obs_|M*) plays an important role in Bayesian model selection [11], [17], [37], [38]. The marginal likelihood is represented as
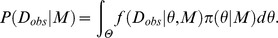
In Bayesian model selection, a model *M* is a variable. To compare models *M*_1_ and *M*_2_, we can use the Bayes factor [37] represented as
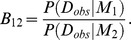
From the Bayes' theorem, the Bayes factor is the ratio of posterior odds and prior odds as follows [37]:
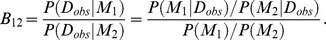

*P*(*M*) is the prior probability of the model *M*. *P*(*M|D_obs_*) is the posterior probability of the model *M*. When the prior probabilities of the competing models are equal (*P*(*M*_1_) = *P*(*M*_2_)), the Bayes factor equals to the ratio of the posterior probabilities
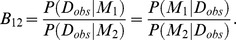
Depending on the Bayes factor, we can conduct Bayesian model selection [15], [17], [37], [38].

To conduct Bayesian model selection in the ABC framework, we need to define the marginal likelihood in the ABC framework. For definition, we start from the augmented posterior probability of a model represented as
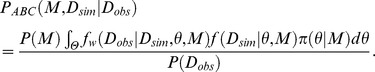
On another front, we have the relationship
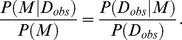
The numerator of the right hand side of the equation corresponds to the conventional marginal likelihood. We can obtain the similar relationship with the augmented posterior distribution as
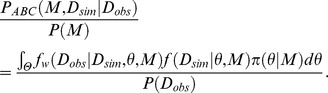
Thus, we can assume the marginal likelihood in the ABC framework as
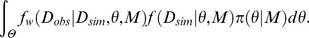
The validity of the approximation can be confirmed by setting the weighting function to the indicator function as
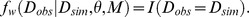
The indicator function equals to 1 if the observed data equals to the simulated data and 0 if not. By this setting, the marginal likelihood in the ABC framework is consistent with the conventional marginal likelihood as follows:
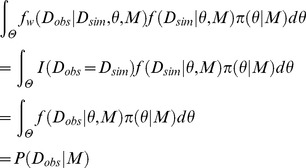
Thus, if we set the weighting function to the indicator function as
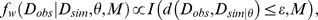
and *ε* is small enough, the marginal likelihood in the ABC framework is a good approximation of the conventional marginal likelihood. This is same as the case of the augmented posterior distribution for parameter inference in the ABC framework. Therefore, the Bayes factor can be defined and calculated in the ABC framework as
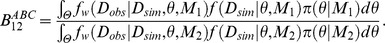
Depending on the Bayes factor *B^ABC^_12_*, we can conduct Bayesian model selection in the ABC framework.

### Population annealing: algorithm

Population annealing [28], [29] is one of the population Monte Carlo algorithm [30] as same as SMC. Thus, in principle, population annealing can avoid its sampler being trapped in a low probability area as same as SMC [23]. Although population annealing is usually used in statistical mechanics to sample from a canonical distribution [28], [31], we show that population annealing can be used to compute Bayesian posterior distributions.

Population annealing uses particles to approximately represent a target probability distribution. Each particle has one set of concrete values of a multidimensional variable *x*, which corresponds to a sample from the target distribution. For population annealing, we define the intermediate distributions *f_IM_*(*x*) as same as annealed importance sampling [39]. The first intermediate distribution *f_IM_^0^*(*x*) is set to the probability distribution of which sampling is easy. The last intermediate distribution *f_IM_^N^*(*x*) is set to the target distribution i.e. Bayesian posterior distribution in Bayesian approach. In population annealing, the first intermediate distribution is gradually changed to the last intermediate distribution through a number of intermediate distributions *f_IM_^n^*(*x*) (0<*n*<*N*) between the first one (*n* = 0) and the last one (*n = N*). In the case of statistical mechanics, the intermediate distributions are canonical distributions with different temperatures of the system. Thus, annealing is directly corresponds to the gradual decrease of temperature of canonical distributions. Application to Bayesian statistics mimics the process [39]. In population annealing, particles are moved and their weights are changed to follow the intermediate distribution in each annealing step. Population annealing algorithm proceeds as follows [28], [29]:

**PA1.** Generate *x* ^k^∼*f_IM_^0^*(*x*) (1**≦** *k* **≦** *K*) independently and set the initial value of weight to *w_0_* ^k^ = 1/*K*, where *K* is the total number of particles.

**PA2.** Repeat the following procedure from *n* = 1 to *n* = *N* for each particle independently.

- Update weights by following equation
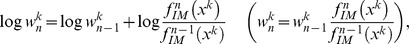
and normalize.
- Update *x* ^k^ independently by finite number of MCMC movements as the stationary distribution is consistent with the *n*-th intermediate distribution *f_IM_^n^*(*x*).
- Resample particles at appropriate timing by following procedure. For each particle, set the probability as
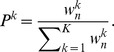
Sample new particles with replacement following the probabilities and set the weight as *w_n_* ^k^ = 1/*K*.
- Set *n* = *n*+1 and return to (a).

In the PA algorithm, we transposed the MCMC process and the resampling process in the original PA algorithm [28]. As MCMC process in PA2 (b), we use the Metropolis-Hastings algorithm (Text S1) [40], [41]. To use population annealing in the ABC framework, we need to use ABC-MCMC (See Text S1) in PA2 (b) [22], [24]. To decide the appropriate timing of resampling in PA2 (c), we used effective sample size (ESS) as same as Sisson et al's study [23]. ESS is defined as
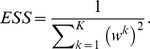
ESS represents the bias of particles' weights. Particles are resampled when the ESS value falls below a threshold. In this study, we set the threshold to *K*/2. The output of the PA algorithm is the *K* particles which each of them has *x* ^k^ associated with weight *w_N_* ^k^ (1≦*k*≦*K*). These weights are set to follow the target distribution *f_IM_^N^*(*x*). By counting *x* ^k^ associated with weights *w_N_* ^k^, we can obtain the target distribution *f_IM_^N^*(*x*).

### Population annealing: application to Bayesian parameter inference

Population annealing can be used to conduct Bayesian parameter inference. In the case of parameter inference, a multidimensional variable *x* corresponds to a set of parameters *θ* and a fixed model *M*. The target distribution for sampling is the posterior distribution of parameters *π*(*θ*|*D_obs_*,*M*) (See Text S2 as a example based on [17], [39]) or the augmented posterior distribution *π_ABC_*(*θ,D_sim_|D_obs_*,*M*) in the ABC framework. In the ABC framework, we firstly set the weighting function to the indicator function. Then we can define the intermediate distribution as follows:
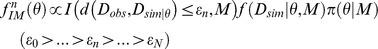
As the tolerance *ε_n_* decreases, the intermediate distribution gradually changes from the first intermediate distribution (*ε = ε_0_*) to the last intermediate distribution (*ε = ε_N_*) corresponding to the augmented posterior distribution of parameters as the similar manner in ABC-SMC [15], [18]. The concrete schedule of *ε_n_* is set depending on the problem.

### Population annealing: application to Bayesian model selection

Population annealing can be used to conduct Bayesian model selection in the ABC framework. We firstly set the weighting function to the indicator function. Under this setting, with a large number *K*, the marginal likelihood in the ABC framework can be approximated as follows:
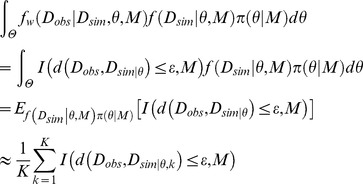

*E*[·] indicates an expectation value. *D_sim|θ,k_* is a sample from *f*(*D_sim_*|*θ,M*)*π*(*θ|M*). Thus, under a fixed total number of particles *K*, the number of particles satisfying the condition *d*(*D_obs_,D_sim_*)**≦** *ε* is proportional to the marginal likelihood in the ABC framework. Population annealing enables us to count the number of particles satisfying the condition. Because we use the indicator function, each particle has a common value of weight (non-zero weight) or zero weight in population annealing. Thus, starting with a fixed total number of particles *K*, the number of particles which have non-zero weight at the end of population annealing is proportional to the marginal likelihood in the ABC framework. This is because the particles at the end of the algorithm follow the last intermediate distribution corresponding to the target distribution
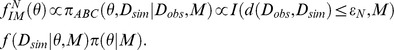
However, even though starting with a fixed total number of particles, resampling processes recover the number of non-zero weight particles to the total number of particles *K*. Thus, each time of resampling, the number of non-zero weight particles before resampling *k_resample_* should be memorized. By multiplying *k_resample_*/*K* to the number of particles at the end of the algorithm, we can estimate the marginal likelihood in the ABC framework. In this study, we adopted this solution. This solution is based on the similar idea of the “ABC-SMC approximation of the marginal likelihood” [18]. Another solution against resampling is to eliminate the resampling process from population annealing. In this study, we did not adopt that solution. This is because the resampling process is considered to be important to avoid the fluctuation of the weight factor and conduct stable calculations [28].

The computation of the number of particles satisfying the condition *d*(*D_obs_,D_sim_*)≦*ε* can be also done by ABC rejection sampler (Text S1) [15], [23], [24], [42]. Fixing the total number of sampling trials, the number of accepted particles in ABC rejection sampler is proportional to the marginal likelihood in the ABC framework. However, it is known that the acceptance rate often gets lower in the case that the prior distribution is very different from the posterior distribution [15]. We can avoid the problem by population annealing, because the intermediate distributions gradually changes from the prior distribution to the posterior distribution in population annealing.

### Simulations with the posterior parameter ensemble

In this study, instead of sampling a number of parameters from the posterior distribution, we ran the simulations with all the output particles of population annealing. This is fundamentally same as the simulations with the posterior parameter samples [11], [13]. In population annealing, each particle at the end of the algorithm (output particles of population annealing) has concrete values of parameters *θ* ^k^ associated with weight *w_N_* ^k^ (1≦*k*≦*K*). These weights are set to follow the target posterior distribution. Thus, the simulations with *θ* ^k^ weighted by *w_N_* ^k^ for all the *K* particles correspond to the simulations with the parameter ensemble sampled from the posterior distribution. Practically, this can be done with use of the same particles used to obtain the posterior distribution of parameters. In this manner, population annealing is a convenient algorithm to generate the posterior parameter ensemble and conduct the ensemble simulation.

## Results

### Flow of the tests

In this section, we explain the flow of the tests which start from the next section. To test the validity of our method, we applied our method to the two test problems. In the first test, we introduced two mathematical models of the feed-forward loop network motif [32]–[36] in the next “Mathematical models and simulation setting of the feed-forward loop network motif” section. Then, we artificially generated the observed data from one of the models with the given answer values of parameters. Next, in the “Parameter inference, reproduction and prediction of the dynamics of the feed-forward loop models” section, we firstly tried to estimate the answer values of parameters by computing the posterior distribution of parameters. Secondly, we tested whether the simulations with posterior parameter ensemble can reproduce the data used for parameter inference. In addition, we tested whether the simulations with posterior parameter ensemble can correctly predict the newly generated data which was not used for parameter inference. Next, in the “Model selection between the feed-forward loop models” section, we tested whether the true model that the observed data was generated from was correctly selected or not by computing the Bayes factor. Lastly, in the remaining sections, we showed the results of the second test. In the second test, we used the insulin dependent AKT pathway model [47], [48]. In the test, we tried to reproduce the experimental time-series data of phosphorylated AKT. We also conducted Bayesian model selection between the wild type AKT pathway model and the mutant AKT pathway model. Importantly, we used the open experimental data in the second test.

### Mathematical models and simulation setting of the feed-forward loop network motif

#### Mathematical models of the feed-forward loop network motif

**Figure 1:**
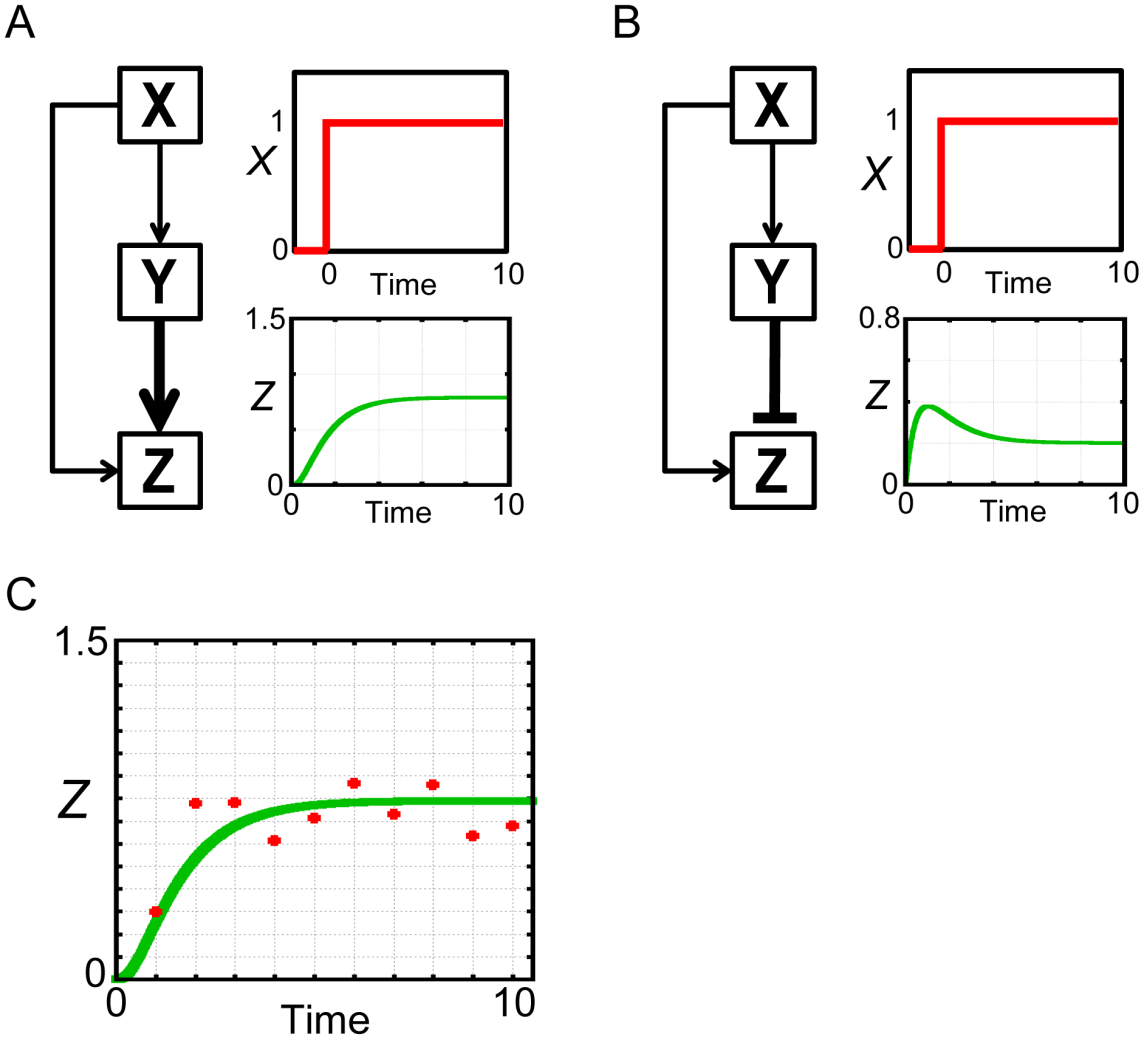
Feed-forward loop network motif models. (A) Structure and dynamics of the coherent FFL model in response to the step stimulation of *X*. (B) Structure and dynamics of the incoherent FFL model in response to the step stimulation of *X*. (C) Generated observed data. The observed data (red points) was generated from the coherent FFL model by adding observation noise into the green-colored trajectory. The green-colored trajectory is same as that of in (A).

For the first test, we used the mathematical models of the feed-forward loop network motif [32]–[36]. Network motifs are building blocks of transcription network found in diverse organisms [34]. One of the significant network motifs is the feed-forward loop (FFL), which consists of three components, *X*, *Y* and *Z*. There are totally eight possible structures of FFL, which can be divided into coherent FFL or incoherent FFL [33], [34]. In addition, there are AND logic and OR logic for the activation of *Z* by *X* and *Y* [33], [34]. In the current test study, we used one of the coherent FFL and one of the incoherent FFL with AND logic (Figure 1.A, B). Their dynamics can be represented by the following ordinary differential equations [33].

*Coherent FFL*

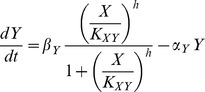


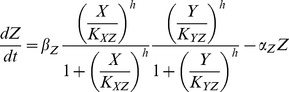



*Incoherent FFL*

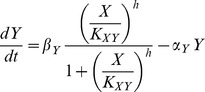


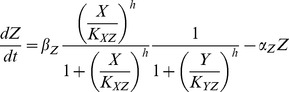
In the equations, *X* is an input signal, set to a step function or a pulse in the test. *Y* and *Z* are variables. Each model contains eight parameters, *α_Y_*, *α_Z_*, *β_Y_*, *β_Z_*, *K_XY_*, *K_XZ_*, *K_YZ_* and *h*. In this test study, we did not specifically define the units of these parameters and time for simplicity.

#### Generation of the artificial observed data

To use in the test, we artificially generated the observed data from the coherent FFL model (Figure 1.A). To generate the data, we firstly set the answer values of parameters *θ_answer_* = (*α_Y_*, *α_Z_*, *β_Y_*, *β_Z_*, *K_XY_*, *K_XZ_*, *K_YZ_*) = (1, 1, 1, 1, 0.1, 0.1, 0.5). In parameter inference, we tried to infer these seven values. In model selection, these seven parameters were free parameters. Remaining Hill coefficient was set to *h* = 2. This value was always given and fixed in this test study. With these answer values and the fixed value of Hill coefficient, the coherent FFL and the incoherent FFL models showed distinct dynamics of *Z* in response to the step stimulation of *X* (Figure 1.A, B). Secondly, we added Gaussian noise with mean 0 and variance 0.01 as observation noise to the time-series data of *Z* in Figure 1.A. The generated data was represented as *D_obs_* = {*Z^t^_obs_*, t = 1∼10} (red points in Figure 1.C, concrete values are shown in Table S1). The observed data *D_obs_* was used for all the computations of parameter inference and model selection in the first test with FFL models.

#### Prior distribution and weighting function

For Bayesian parameter inference and model selection, we set the prior distribution of each parameter to independently follow the uniform distribution on a common logarithmic scale. Logarithmic scale is often used in systems biology [13], [14], [43], [44]. The upper-bound and the lower-bound of each uniform distribution were set to 10-folds value and 1/10-folds value of each answer value of parameter respectively i.e. log_10_ *α_Y_* ∼ *U*[0.1,10], log_10_ *α_Z_* ∼ *U*[0.1,10], log_10_ *β_Y_* ∼ *U*[0.1,10], log_10_ *β_Z_* ∼ *U*[0.1,10], log_10_ *K_XY_* ∼ *U*[0.01,1], log_10_ *K_XZ_* ∼ *U*[0.01,1], log_10_ *K_YZ_* ∼ *U*[0.05,5]. Here, *U* represents the uniform distribution.

In this test study, we set the weighting function to the indicator function [14], [22]–[24], [26] represented as
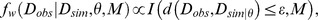
and set the distance *d*(*D_obs_,D_sim|θ_*) as
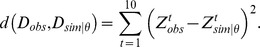

*D_sim|θ_* = {*Z^t^_sim_*_|*θ*_, t = 1∼10} is a simulated time-series data of *Z* with *θ*.

#### Numerical simulation

The total number of particles in population annealing was set to *K* = 100000. As the default annealing schedule, the tolerance was gradually decreased as follows:
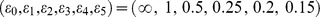
The proposal distribution of ABC-MCMC in population annealing was set to the uniform distribution on a common logarithmic scale. In concrete terms, at each step of ABC-MCMC, one of the parameters was randomly chosen, and the uniform random number between −0.25 to 0.25 was added on a common logarithmic scale. For each particle, ABC-MCMC movements in population annealing were set to 7 steps. This is the number of the inferred parameters.

For time-series calculations, the ordinary differential equations were numerically solved by the fourth-order Runge-Kutta method with a time step of 0.01. Both of the initial amounts of *Y* and *Z* were set to 0 in all the calculations. *X* was set to the step function (*X* = 1 during the entire simulation) or the pulse (*X* = 1 until time = 5, then *X* = 0).

### Parameter inference, reproduction and prediction of the dynamics of the feed-forward loop models

#### Coherent FFL model

**Figure 2:**
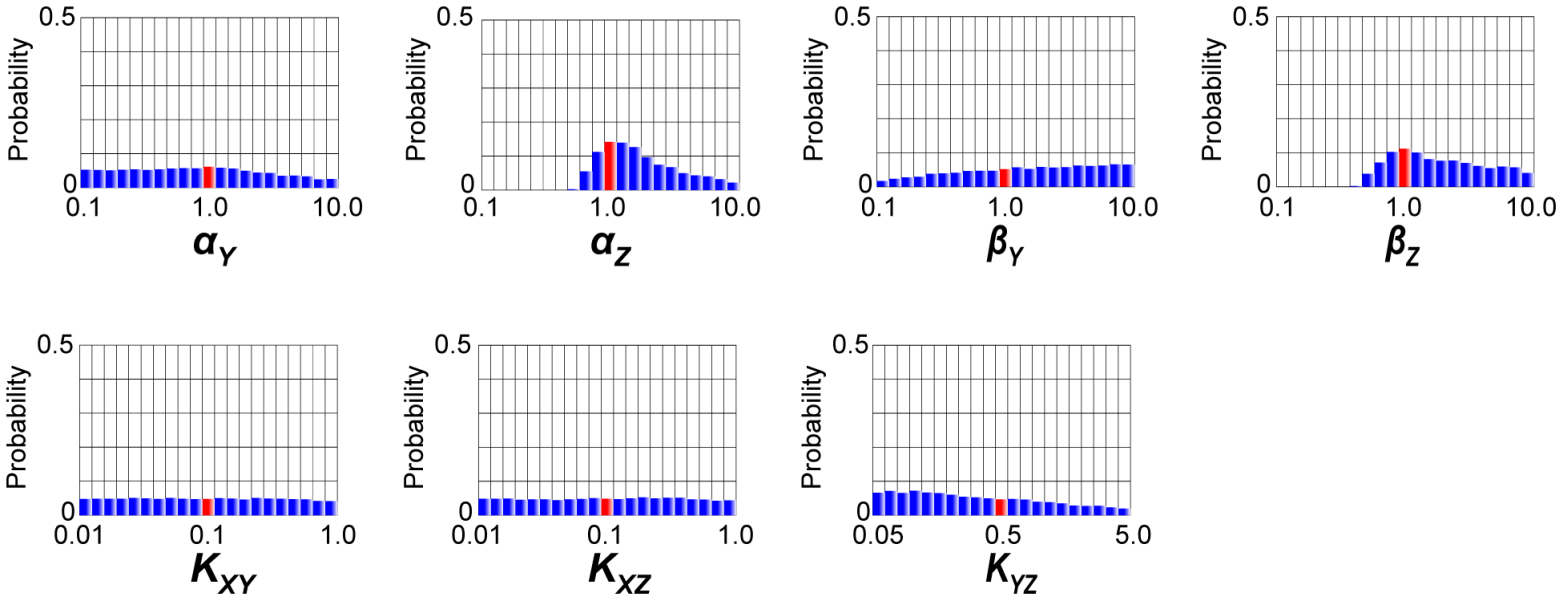
Marginal probability distributions of the parameters in the coherent FFL model. The joint probability distribution approximated by 100000 particles was marginalized for each parameter. Red-colored classes represent the “answer” value of each parameter.

Firstly, we conducted parameter inference of the coherent FFL model by population annealing. The computed joint posterior distribution of the inferred parameters was marginalized and shown in Figure 2. In each marginal distribution, the red-colored class corresponds to the answer value of each parameter. In Figure 2, *α_Z_* and *β_Z_* seemed to be inferred well to some extent compared to other parameters. However, marginal distributions of other parameters were almost similar to the uniform distributions, which are same as the prior distributions in this study. If our purpose of parameter inference is the estimation of the correct values of unknown parameters with high credibility, we failed in accomplishing our purpose in this case.

**Figure 3:**
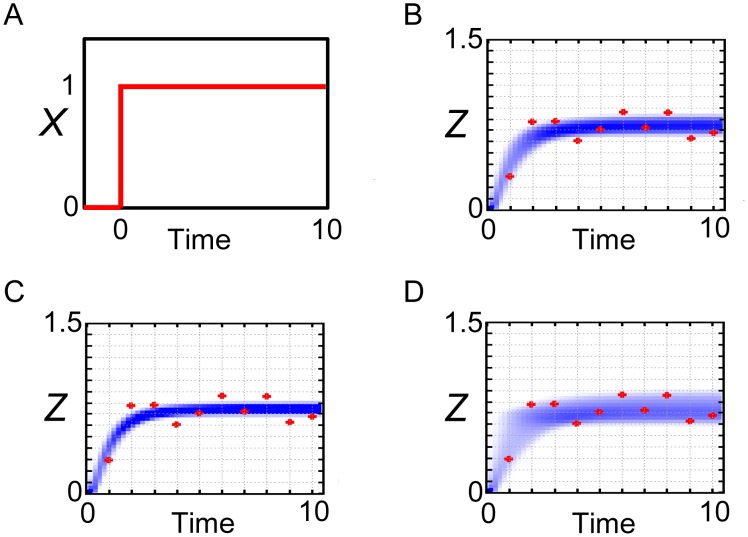
Reproduction of the observed data by the coherent FFL model. Simulations with the posterior parameter ensemble of the coherent FFL model in response to the step stimulation of *X*. (A) Input step stimulation of *X*. (B) Annealing schedule: (*ε_0_, ε_1_, ε_2_, ε_3_, ε_4_, ε_5_*) = (∞, 1, 0.5, 0.25, 0.2, 0.15). (C) Annealing schedule: (*ε_0_, ε_1_, ε_2_, ε_3_, ε_4_, ε_5_*) = (∞, 1, 0.5, 0.25, 0.15, 0.11). (D) Annealing schedule: (*ε_0_, ε_1_, ε_2_, ε_3_, ε_4_, ε_5_*) = (∞, 2, 1, 0.75, 0.5, 0.25). Blue-colored area is the probability density consists of the simulated trajectories. Red points are the observed data.

However, if our purpose is the reproduction or the prediction of the system dynamics, instead of choosing one set of representative values of parameters, we can run the simulations with the posterior parameter ensemble. In this study, we ran the simulations with use of all the 100000 output particles (parameter sets) computed by population annealing. The simulations with posterior parameter ensemble give the probability density consists of the simulated trajectories. Reproductions of the observed data used for parameter inference were shown in Figure 3. We ran the simulations in response to the step stimulation of *X* (Figure 3.A) as same as the generation process of the observed data. In Figure 3.B, the area of the probability density consists of the simulated trajectories (blue-colored area) could capture the observed data used for parameter inference (red points). When the annealing schedule was changed to the smaller tolerances (*ε_0_, ε_1_, ε_2_, ε_3_, ε_4_, ε_5_*) = (∞, 1, 0.5, 0.25, 0.15, 0.11), the area of the probability density got narrower (Figure 3.C). When the annealing schedule was changed to the larger tolerances (*ε_0_, ε_1_, ε_2_, ε_3_, ε_4_, ε_5_*) = (∞, 2, 1, 0.75, 0.5, 0.25), the area of the probability density got broader and captured all the red points (Figure 3.D). As shown, the area of the probability density differs depending on the annealing schedules. This result is unsurprising because the last *ε* values strongly restrict the acceptable trajectories to the observed data. Thus, we are recommended to try a number of annealing schedules to check the influence of the schedules on the simulated data. In either case, the posterior parameter ensemble could reproduce the observed data well.

**Figure 4:**
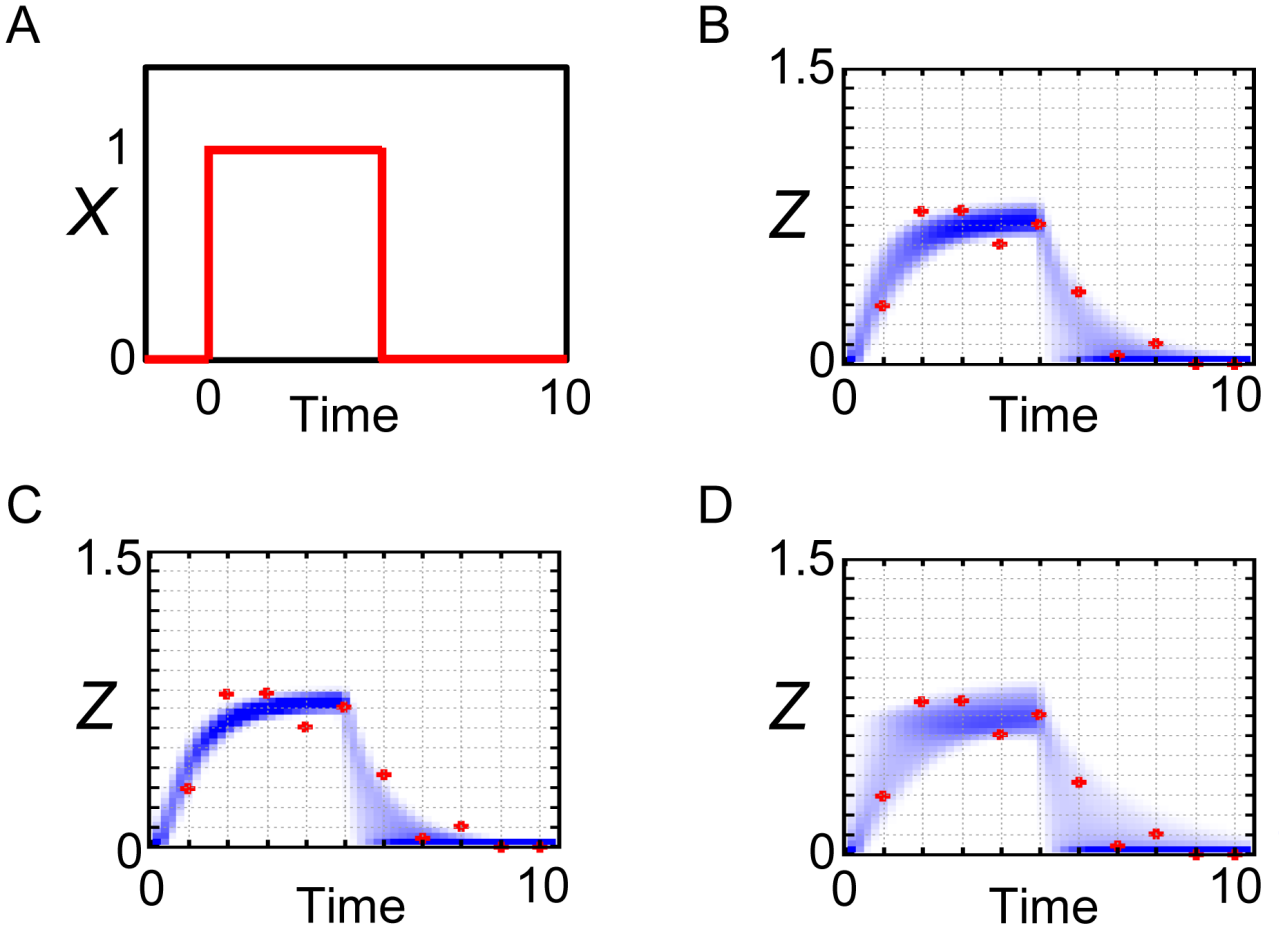
Prediction of the observed data by the coherent FFL model. Simulations with the posterior parameter ensemble of the coherent FFL model in response to the pulse stimulation of *X*. (A) Input pulse stimulation of *X*. (B) Annealing schedule: (*ε_0_, ε_1_, ε_2_, ε_3_, ε_4_, ε_5_*) = (∞, 1, 0.5, 0.25, 0.2, 0.15). (C) Annealing schedule: (*ε_0_, ε_1_, ε_2_, ε_3_, ε_4_, ε_5_*) = (∞, 1, 0.5, 0.25, 0.15, 0.11). (D) Annealing schedule: (*ε_0_, ε_1_, ε_2_, ε_3_, ε_4_, ε_5_*) = (∞, 2, 1, 0.75, 0.5, 0.25). Blue-colored area is the probability density consists of the simulated trajectories. Red points are the observed data.

Furthermore, we examined whether the simulations with the posterior parameter ensemble can capture the data which was not used for parameter inference. We newly generated the observed data of *Z* (red points in Figure 4, concrete values are shown in Table S2) in response to the pulse stimulation of *X* (Figure 4.A) adding the same Gaussian noise in Figure 1.C. Then we ran the simulations in response to the pulse stimulation of *X* with use of all the 100000 particles which were the output of population annealing. In Figure 4.B, the probability density consists of the simulated trajectories could capture and predict the observed data which was not used for parameter inference. In addition, when the annealing schedule was changed to the smaller tolerances (*ε_0_, ε_1_, ε_2_, ε_3_, ε_4_, ε_5_*) = (∞, 1, 0.5, 0.25, 0.15, 0.11) (Figure 4.C) or the larger tolerances (*ε_0_, ε_1_, ε_2_, ε_3_, ε_4_, ε_5_*) = (∞, 2, 1, 0.75, 0.5, 0.25) (Figure 4.D), the areas of the probability densities could capture and predict the observed data with different rigors.

As a whole, the simulations with the posterior parameter ensemble could, not only reproduce the data used for parameter inference, but also capture and predict the data which was not used for parameter inference. Because the posterior parameter ensemble corresponds to the output particles of population annealing, population annealing is an efficient and convenient algorithm for the simulations with posterior parameter ensemble.

#### Incoherent FFL model

**Figure 5:**
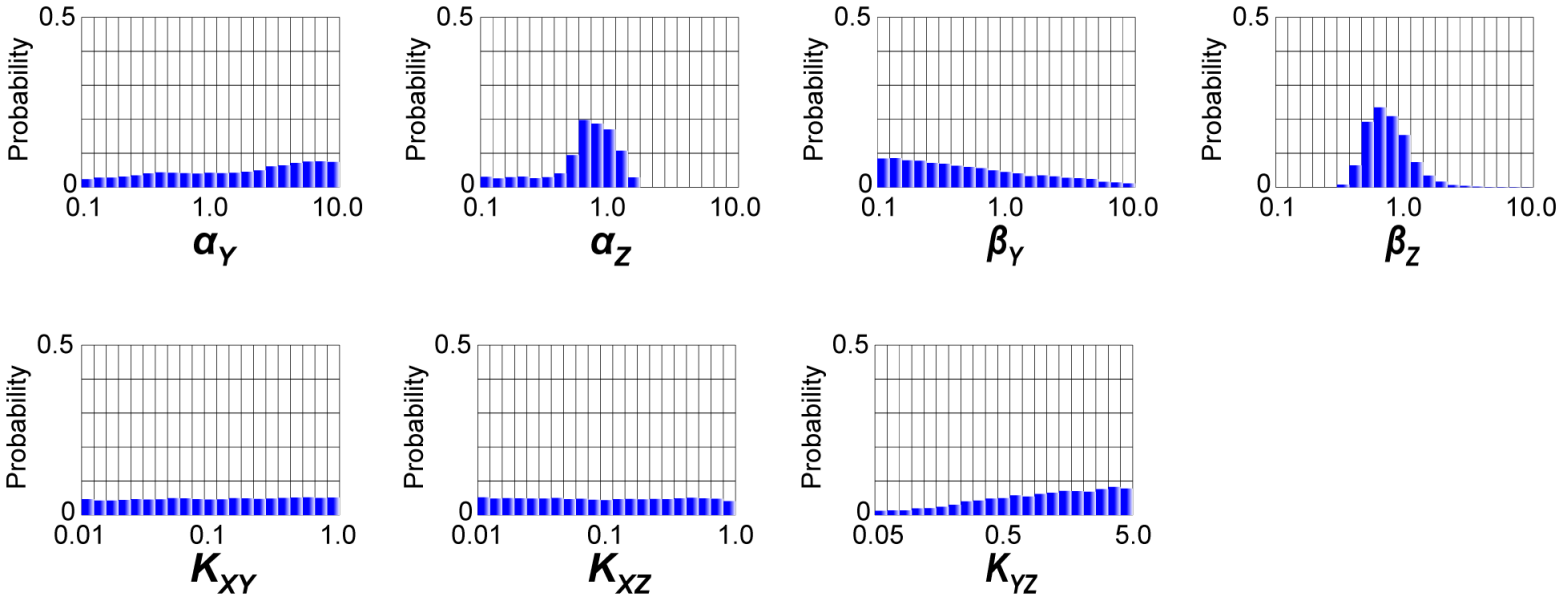
Marginal probability distributions of the parameters in the incoherent FFL model. The joint probability distribution approximated by 100000 particles was marginalized for each parameter.

Next, we conducted parameter inference of the incoherent FFL model by population annealing. Note that parameter inference was conducted with the observed data generated from the coherent FFL model in Figure 1.C, not from the incoherent FFL model. The joint posterior distribution of the inferred parameters was marginalized and shown in Figure 5. We can see the distinct tendencies of marginal distributions between the coherent FFL model and the incoherent FFL model. For example, the marginal distribution of *α_Z_* had a tail in smaller values. This is opposite to that of the coherent FFL (Figure 2). *β_Y_* and *K_YZ_* also showed the opposite tendency between the incoherent FFL model and the coherent FFL model (Figure 2). These differences were considered to come from the difference of the interaction from *Y* to *Z* (Figure 1.A, B). Although there were these kinds of small differences, most of the marginal posterior distributions of the incoherent FFL model were similar to the uniform distributions. This result is similar to the result of the coherent FFL model.

**Figure 6:**
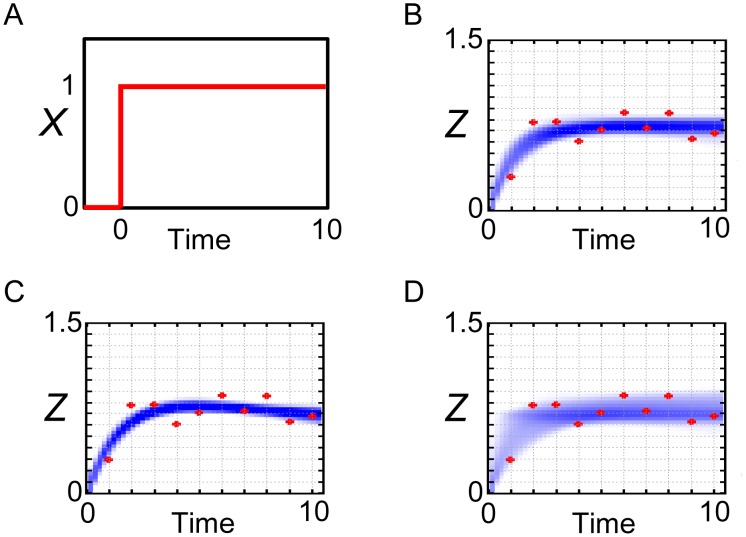
Reproduction of the observed data by the incoherent FFL model. Simulations with the posterior parameter ensemble of the incoherent FFL model in response to the step stimulation of *X*. (A) Input step stimulation of *X*. (B) Annealing schedule: (*ε_0_, ε_1_, ε_2_, ε_3_, ε_4_, ε_5_*) = (∞, 1, 0.5, 0.25, 0.2, 0.15). (C) Annealing schedule: (*ε_0_, ε_1_, ε_2_, ε_3_, ε_4_, ε_5_*) = (∞, 1, 0.5, 0.25, 0.15, 0.11). (D) Annealing schedule: (*ε_0_, ε_1_, ε_2_, ε_3_, ε_4_, ε_5_*) = (∞, 2, 1, 0.75, 0.5, 0.25). Blue-colored area is the probability density consists of the simulated trajectories. Red points are the observed data.

In addition, as shown in Figure 6.B, C and D, the simulations with the posterior parameter ensemble of the incoherent FFL model in response to the step stimulation of *X* (Figure 6.A) showed similar results to those of the coherent FFL model (Figure 3). We again note that the observed data used for parameter inference was generated from the coherent FFL model, not from the incoherent FFL model. This result indicates that, by setting the values of parameters suitably, even the false model (incoherent FFL model) can reproduce the observed data with comparable level to the true model (coherent FFL model). This result also emphasizes the importance of the concrete values of kinetic parameters for the dynamics of the system [45], not only the network structures. One attention point is the case of the annealing schedule (*ε_0_, ε_1_, ε_2_, ε_3_, ε_4_, ε_5_*) = (∞, 1, 0.5, 0.25, 0.15, 0.11) (Figure 6.C). In this case, the probability density became slightly parabolic. This might be a kind of over-fitting to the data.

**Figure 7:**
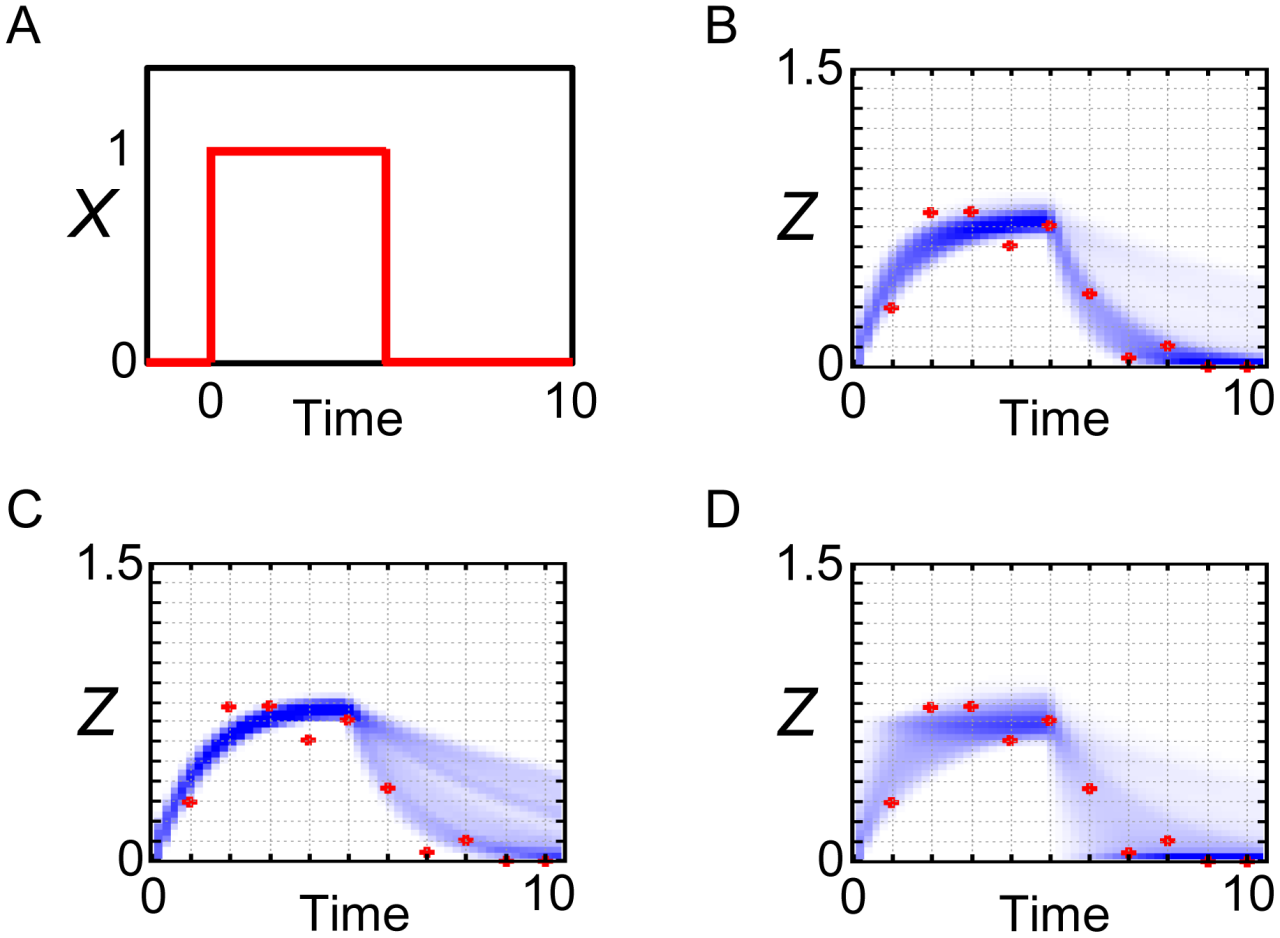
Prediction of the observed data by the incoherent FFL model. Simulations with the posterior parameter ensemble of the incoherent FFL model in response to the pulse stimulation of *X*. (A) Input pulse stimulation of *X*. (B) Annealing schedule: (*ε_0_, ε_1_, ε_2_, ε_3_, ε_4_, ε_5_*) = (∞, 1, 0.5, 0.25, 0.2, 0.15). (C) Annealing schedule: (*ε_0_, ε_1_, ε_2_, ε_3_, ε_4_, ε_5_*) = (∞, 1, 0.5, 0.25, 0.15, 0.11). (D) Annealing schedule: (*ε_0_, ε_1_, ε_2_, ε_3_, ε_4_, ε_5_*) = (∞, 2, 1, 0.75, 0.5, 0.25). Blue-colored area is the probability density consists of the simulated trajectories. Red points are the observed data.

However, the prediction of *Z* dynamics in response to the pulse stimulation of *X* (Figure 7.A) did not succeed well (Figure 7.B, C and D). After the decrease of *X* at time = 5, part of the trajectories of *Z* could not capture the observed data well. Thus, if we have the observed data in response to the pulse stimulation of *X*, we might be able to naively select the true model (coherent FFL model) in a visual way. However, it is not always possible to obtain the convenient data for model selection. To deal with this kind of problem, we can conduct Bayesian model selection.

### Model selection between the feed-forward loop models

Next, we conducted Bayesian model selection, comparing the coherent FFL model with the incoherent FFL model. To conduct Bayesian model selection, we computed the Bayes factor in the ABC framework represented as
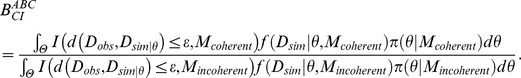
Larger *B^ABC^_CI_* indicates that the coherent FFL model is selected with stronger evidence against the incoherent FFL model. In the current test study, model selection was conducted with the observed data generated from the coherent FFL model (Figure 1.C). Thus, *B^ABC^_CI_* should be a large value, at least larger than 1 to propose the validity of Bayesian model selection by population annealing.

Computation of *B^ABC^_CI_* was done under different annealing schedules in population annealing. Annealing schedules were set to same as those in parameter inference. For each annealing schedule, the mean and the standard deviation of 10 independent computations of *B^ABC^_CI_* were shown in Table 1. In Table 1, as the value of the last *ε* got smaller, *B^ABC^_CI_* got larger. This result indicates that, as the models need to reproduce the observed data more rigorously, the coherent FFL model is selected with stronger evidence against the incoherent FFL model. In addition, *B^ABC^_CI_* was always larger than 1. This result is consistent with the fact that the observed data used for model selection was generated from the coherent FFL model, and the prediction that the coherent FFL model must be selected in this case. Thus, Bayesian model selection by population annealing is valid.

**Table 1:** Bayes factor *B^ABC^_CI_* computed with different annealing schedules in population annealing and different last epsilons in ABC rejection sampler.

annealing schedule/last *ε*	*B^ABC^_CI_* (PA)	*B^ABC^_CI_* (ARS)
AS1/0.25	1.367±0.033	1.391±0.031
AS2/0.15	1.896±0.089	1.935±0.096
AS3/0.11	9.618±1.175	10.079±2.830

Abbreviations are as follows: PA: population annealing, ARS: ABC rejection sampler. AS1: annealing schedule 1 = (∞, 2, 1, 0.75, 0.5, 0.25), AS2: annealing schedule 2 = (∞, 1, 0.5, 0.25, 0.2, 0.15), AS3: annealing schedule 3 = (∞, 1, 0.5, 0.25, 0.15, 0.11).

We also conducted Bayesian model selection by ABC rejection sampler (Text S1). For comparison, the value of *ε* in ABC rejection sampler was set to the same value of the last *ε* of population annealing. The total number of sampling trials were set to 100000, same as the number of particles in population annealing. As shown in Table 1, the means of Bayes factors computed by ABC rejection sampler were similar to those computed by population annealing. In the case of large or middle values of *ε*, the standard deviations of Bayes factors computed by ABC rejection sampler were also similar to those of computed by population annealing. However, in the case of small value of *ε*, the standard deviation of the Bayes factor computed by ABC rejection sampler was larger than that of computed by population annealing. Small standard deviation indicates a stable computational result. Thus, these results demonstrate the efficiency of population annealing for computation of the Bayes factors.

### Mathematical model and simulation setting of the AKT pathway model

#### Mathematical model of the AKT pathway model

For the second test, we focused on the insulin dependent AKT pathway model [47], [48]. This is because the model is more complicated than the FFL models, and the test with the model seems to be difficult. In addition, the experimental data about the pathway are open in Noguchi et al.'s web page [48].

Insulin is an important hormone which regulates various metabolic processes [49]. Especially, regulation of sugar metabolism is a very important role of insulin because defect in insulin action is related to type 2 diabetes mellitus [49]. As an intracellular signal transduction pathway, the AKT pathway plays an important role for the action of insulin [49], [50]. Using a combination of experiments and mathematical modeling, Kubota et al. and Noguchi et al. demonstrated that temporal patterns of insulin selectively control glucose metabolism through the AKT pathway [47], [48].

**Figure 8:**
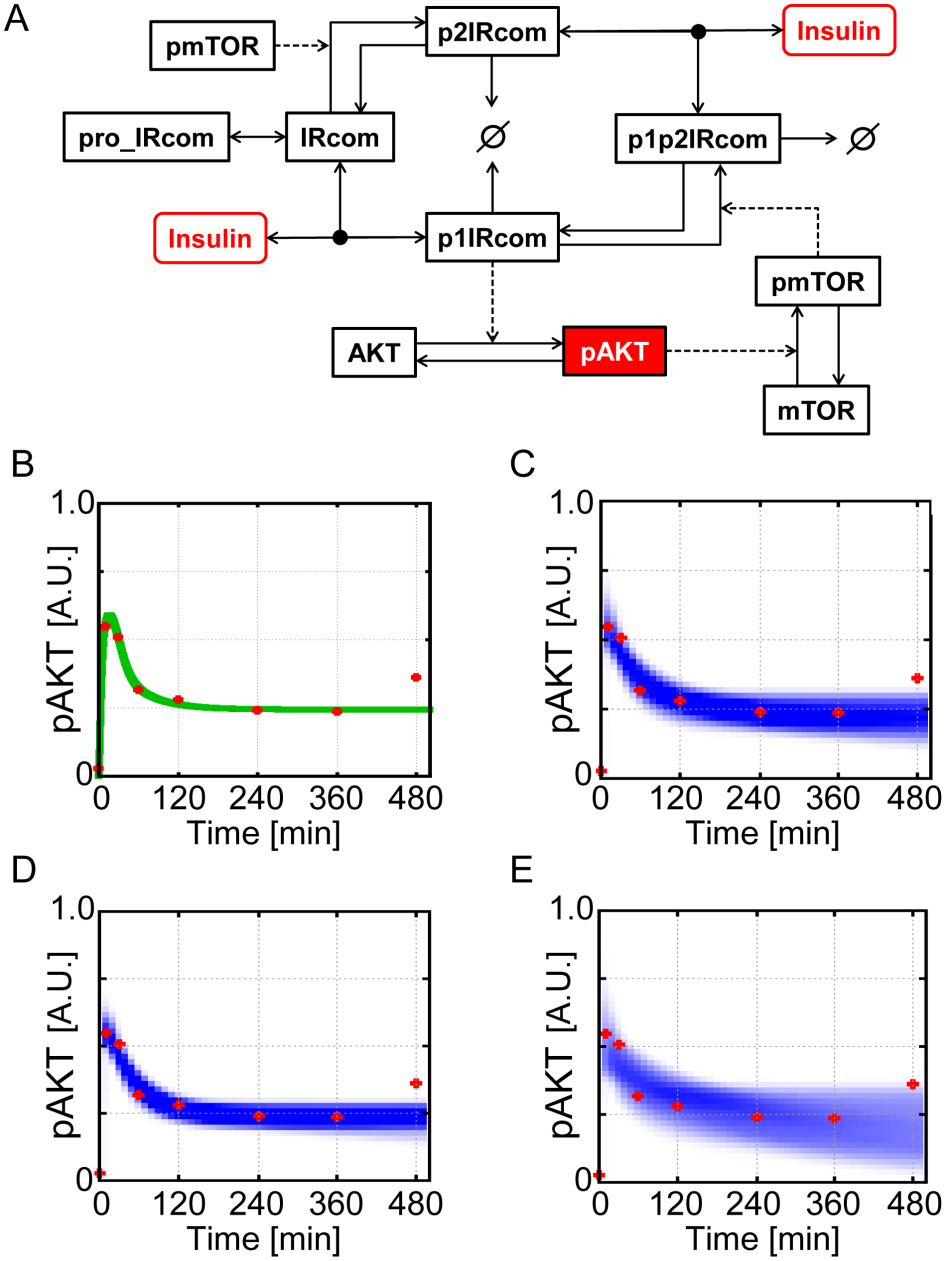
AKT pathway model. (A) Structure of the AKT pathway model. The input signal is insulin. The output signal is pAKT. Solid arrows represent mass flows. Solid arrows directional to a lined circle represent degradation processes. Solid arrows with black circles represent association/dissociation processes. Dotted arrows represent enhancement of the processes. (B) Experimental data (red points) and simulated trajectory (green trajectory) of pAKT in response to the step stimulation of 1 nM insulin. (C), (D), (E) Reproduction of the experimental data. Simulations were run with the posterior parameter ensemble in response to the step stimulation of 1 nM insulin. (C) Annealing schedule: (*ε_0_, ε_1_, ε_2_, ε_3_, ε_4_, ε_5_, ε_6_, ε_7_, ε_8_, ε_9_, ε_10_, ε_11_, ε_12_, ε_13_, ε_14_*) = (∞, 1.5, 1.0, 0.75, 0.5, 0.25, 0.1, 0.09, 0.08, 0.07, 0.06, 0.05, 0.04, 0.03, 0.02). (D) Annealing schedule: (*ε_0_, ε_1_, ε_2_, ε_3_, ε_4_, ε_5_, ε_6_, ε_7_, ε_8_, ε_9_, ε_10_, ε_11_, ε_12_, ε_13_, ε_14_, ε_15_*) = (∞, 1.5, 1.0, 0.75, 0.5, 0.25, 0.1, 0.09, 0.08, 0.07, 0.06, 0.05, 0.04, 0.03, 0.02, 0.01). (E) Annealing schedule: (*ε_0_, ε_1_, ε_2_, ε_3_, ε_4_, ε_5_, ε_6_, ε_7_, ε_8_, ε_9_, ε_10_, ε_11_*) = (∞, 1.5, 1.0, 0.75, 0.5, 0.25, 0.1, 0.09, 0.08, 0.07, 0.06, 0.05). Blue-colored area is the probability density consists of the simulated trajectories. Red points are the experimental data.

For the second test, we decided to use a part of the original insulin dependent AKT pathway models. The original models incorporates additional metabolic pathways downstream of AKT [47], [48]. However, for simplicity, and because the downstream pathways differs between Kubota et al.'s model [47] and Noguchi et al.'s model [48], we employed the insulin-AKT module which is common in the original models. In the employed AKT model (Figure 8.A), the input signal is insulin and the output signal is phosphorylated AKT (pAKT in Figure 8.A). The dynamics of the model is represented by 6 differential equations with 16 rate constants and 9 initial amounts of the components. In this study, we used the same differential equations of the original models [47], [48]. In addition, we used the same values of the initial amounts of components shown in Noguchi et al.'s paper [48]. Remaining 16 rate constants were free parameters in the test.

#### Experimental data of AKT dynamics

A part of the experimental results are open in Noguchi et al.'s web page [48]. In the experimental results, we used the time-series data of pAKT in response to the step stimulation of 1 nM insulin (red points in Figure 8.B) [48]. In their experiments, pAKT level is measured at eight time points (time = 0, 10, 30, 60, 120, 240, 360, 480 minutes) [48]. When the rate constants in the model are set to their default values shown in Noguchi et al.'s paper [48], the simulated trajectory could capture the experimental time-series data of pAKT except for the point at 480 minutes (green trajectory in Figure 8.B). Thus, we decided to use the experimental time-series data of pAKT except for the point at 480 minutes in the test. Those time-series data of pAKT were used for all the computations in the remaining sections.

#### Prior distribution and weighting function

As same as the case of the feed-forward loop network motif models, we set the prior distribution of each parameter to independently follow the uniform distribution on a common logarithmic scale. The upper-bound and the lower-bound of each uniform distribution were set to 10^4^ and 10^−6^ respectively for all the 16 rate constants (parameters) in the model.

In this test study, we set the weighting function to the indicator function [14], [22]–[24], [26] represented as
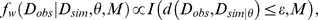
and set the distance *d*(*D_obs_,D_sim|θ_*) as
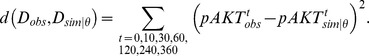

*θ* represents the 16 rate constants in the model. *D_obs_* = {pAKT*^t^_obs_*, t = 0, 10, 30, 60, 120, 240, 360} is the experimental time-series data of pAKT except for the point at 480 minutes in Noguchi et al.'s study [48]. *D_sim|θ_* = {pAKT*^t^_sim_*_|*θ*_, t = 0, 10, 30, 60, 120, 240, 360} is a simulated time-series data of pAKT with *θ*.

#### Numerical simulation

The total number of particles in population annealing was set to *K* = 10000. As the default annealing schedule, the tolerance was gradually decreased as follows:
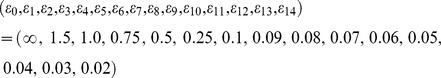
The proposal distribution of ABC-MCMC in population annealing was set to the uniform distribution on a common logarithmic scale. In concrete terms, at each step of ABC-MCMC, one of the parameters was randomly chosen, and the uniform random number between −0.25 to 0.25 was added on a common logarithmic scale. For each particle, ABC-MCMC movements in population annealing were set to 16 steps. This is the number of the free parameters.

For time-series calculations, the ordinary differential equations were numerically solved by the fourth-order Runge-Kutta method with a time step of 0.001 minutes. Initial amounts of the components in the model were set to the values shown in Noguchi et al.'s paper [48]. As the input signal of the model, insulin is set to the step function (Insulin = 1 nM during the entire simulation) in all the computations.

### Reproduction of the experimental time-series data of insulin dependent AKT dynamics

We tested whether the posterior parameter ensemble can capture the experimental time-series data of insulin dependent AKT dynamics. We ran the simulations with use of all the 10000 output particles (parameter sets) of population annealing. Reproductions of the experimental data were shown in Figure 8.C, D, E. In Figure 8.C, the area of the probability density consists of the simulated trajectories (blue-colored area) could capture the experimental time-series data of pAKT (red points). When the annealing schedule was changed to the smaller tolerances (*ε_0_, ε_1_, ε_2_, ε_3_, ε_4_, ε_5_, ε_6_, ε_7_, ε_8_, ε_9_, ε_10_, ε_11_, ε_12_, ε_13_, ε_14_, ε_15_*) = (∞, 1.5, 1.0, 0.75, 0.5, 0.25, 0.1, 0.09, 0.08, 0.07, 0.06, 0.05, 0.04, 0.03, 0.02, 0.01), the area of the probability density got narrower (Figure 8.D). When the annealing schedule was changed to the larger tolerances (*ε_0_, ε_1_, ε_2_, ε_3_, ε_4_, ε_5_, ε_6_, ε_7_, ε_8_, ε_9_, ε_10_, ε_11_*) = (∞, 1.5, 1.0, 0.75, 0.5, 0.25, 0.1, 0.09, 0.08, 0.07, 0.06, 0.05), the area of the probability density got broader (Figure 8.E). In any of these cases, the simulations with the posterior parameter ensemble could reproduce the real experimental data of AKT dynamics, demonstrating the efficiency of our method.

### Model selection of the AKT pathway model

Lastly, we conducted Bayesian model selection of the AKT pathway model. As shown in Figure 8.B, pAKT shows strong transient and weak sustained response, so-called a partial adaptive response. From the network structure of the model, we can predict the mTOR-related negative feedback plays an important role for a partial adaptive response. Thus, for comparison with the wild type model shown in Figure 8.A, we prepared the mutant model which lacks the mTOR-related negative feedback. Lack of the negative feedback was realized by setting the values of related rate constants to zero.

To conduct Bayesian model selection, we computed the Bayes factor in the ABC framework represented as
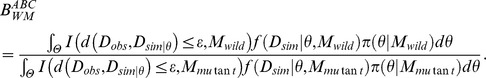
Larger *B^ABC^_WM_* indicates that the wild type model is selected with stronger evidence against the mutant model. If the mTOR-related negative feedback is destroyed, the model will not be able to reproduce a partial adaptive response. Thus, *B^ABC^_WM_* should be a large value, at least larger than 1 to propose the validity of Bayesian model selection by population annealing.

Computation of *B^ABC^_WM_* was done under different annealing schedules in population annealing. Annealing schedules were set to same as those in parameter inference. For each annealing schedule, the mean and the standard deviation of 10 independent computations of *B^ABC^_WM_* were shown in Table 2. In Table 2, the means of *B^ABC^_WM_* were always larger than 1 independent of the annealing schedules. This result indicates that the wild type model was selected with stronger evidence against the mutant model independent of the annealing schedules. This is consistent with the predictions that the mTOR-related negative feedback is important for a partial adaptive response of pAKT, and the wild type model must be selected in this case. These results demonstrate the validity of Bayesian model selection by population annealing.

**Table 2:** Bayes factor *B^ABC^_WM_* computed with different annealing schedules in population annealing and different last epsilons in ABC rejection sampler.

annealing schedule/last *ε*	*B^ABC^_WM_* (PA)	*B^ABC^_WM_* (ARS)
AS1/0.05	1.567±0.496	2.600±1.750
AS2/0.02	2.650±0.959	-
AS3/0.01	1.895±0.933	-

Abbreviations are as follows: PA: population annealing, RS: ABC rejection sampler. AS1: annealing schedule 1 = (∞, 1.5, 1.0, 0.75, 0.5, 0.25, 0.1, 0.09, 0.08, 0.07, 0.06, 0.05), AS2: annealing schedule 2 = (∞, 1.5, 1.0, 0.75, 0.5, 0.25, 0.1, 0.09, 0.08, 0.07, 0.06, 0.05, 0.04, 0.03, 0.02), AS3: annealing schedule 3 = (∞, 1.5, 1.0, 0.75, 0.5, 0.25, 0.1, 0.09, 0.08, 0.07, 0.06, 0.05, 0.04, 0.03, 0.02, 0.01).

We also conducted Bayesian model selection by ABC rejection sampler (Text S1). For comparison, the value of *ε* in ABC rejection sampler was set to the same value of the last *ε* of population annealing. The total number of sampling trials were set to 10000, same as the number of particles in population annealing. As shown in Table 2, ABC rejection sampler could compute the Bayes factor in the case of large value of *ε*. However, the standard deviation was larger than that of computed by population annealing. In addition, in the cases of middle or small value of *ε*, the number of acceptable particle in each independent run was mostly zero. In these cases, we could not calculate the Bayes factors (“-” in Table 2). These results demonstrate the efficiency of population annealing for computation of the Bayes factors.

## Discussion

In this paper, we introduced Bayesian model selection and parameter inference by population annealing. Firstly, we showed that population annealing can be used to compute the Bayesian posterior distributions. Secondly, we showed that the simulations with the posterior parameter ensemble could reproduce the artificial observed data and the experimental data used for parameter inference. In addition, the simulations with the posterior parameter ensemble could capture and predict the observed data which was not used for parameter inference. For both reproduction and prediction, population annealing enables us to run the simulations with the posterior parameter ensemble. These results also emphasize the importance to consider the ensemble or samples of parameters from the posterior distributions for parameter inference and subsequent simulations [11], [13]. Lastly, we showed that the true model was correctly selected by computing the Bayes factor in the test with the FFL models. In the test with the AKT pathway model, the wild type model was correctly selected as expected. In addition, compared to ABC rejection sampler, population annealing showed smaller standard deviations of the Bayes factors. These results indicate that population annealing provides more stable computational result of the Bayes factor than ABC rejection sampler. All of these results support the efficiency of population annealing for Bayesian model selection, parameter inference and subsequent simulations with the parameter ensemble.

In the first test with the FFL models, we used the time-series data consists of 10 time points of *Z*. In the second test with the AKT pathway model, we used the time-series data consists of 7 time points of pAKT. These data scales may seem to be small. Our results may seem to be influenced by the smallness of the data scale. However, the results with the small data were not so largely different from the results obtained with the large data in this study (Text S3, Figure S1). These results indicate that, even though the available data scale is small, our approach can give us reasonable computational results. This is very important for real data analysis because it is not always possible to obtain the large experimental data.

In parameter inference of the FFL models, most of the marginal distributions of parameters were almost similar to the uniform distributions, which were same as the prior distributions in this study. If our purpose of parameter inference is the estimation of the correct values of parameters with high credibility, we should conclude the parameter inference was failed in this case. However, if our purpose is the reproduction or the prediction of the system dynamics, instead of choosing one set of representative values of parameters, we can run simulations with the posterior parameter ensemble. In this study, we want to propose the validity of this approach.

In the simulations with the posterior parameter ensemble, the area of the probability density consists of the simulated trajectories differs among the annealing schedules. This is because the last *ε* values restrict the acceptable trajectories to the observed data. In both of the tests, we set the distance between the observed data and the simulated data as the sum of squared errors. In this case, the computation in the ABC framework is comparable to the maximum-likelihood estimation that errors are assumed to follow Gaussian distribution. This is because minimizing the distance (the smallest *ε* wherever possible) is equivalent to maximizing the Gaussian likelihood function, as pointed out by Toni et al [15]. However in the ABC framework, we can compute, not the maximum-likelihood estimate as a point, but the posterior distribution as a distribution. This allows us to examine the interactions among parameters [21] and to run the simulations with the posterior parameter ensemble. This seems to be an advantage of ABC. In addition, we can change the annealing schedules in population annealing. This allows us to control the rigor of reproduction of the observed data or experimental data flexibly. Although we need to check the influence of the annealing schedules on the simulated data, flexibility of annealing schedule is one of the advantages of population annealing.

In model selection, ABC rejection sampler showed larger standard deviations than those computed by population annealing (Table 1, 2). In addition, ABC rejection sampler could not compute the Bayes factors in some cases (Table 2). As is known, the acceptance rate of ABC rejection sampler often gets lower in the case that the prior distribution is very different from the posterior distribution [15]. This was reconfirmed in this study, because larger standard deviations were obtained when *ε* was smaller (Table 1), which strongly restricts the distributions of parameters. On the other hand, population annealing can avoid the problem and provide the stable computational result of the Bayes factor. This is because the intermediate distributions gradually changes from the prior distribution to the posterior distribution in population annealing.

In this study, with use of the indicator function, we could estimate the marginal likelihood in the ABC framework by population annealing. However, this is valid when the number of non-zero weight particles is proportional to the marginal likelihood. Thus, when the premise is not satisfied, this method cannot be applied. For example, this is the case that the likelihood function is assumed as Gaussian distribution, not in the ABC framework. However, as the solution of this problem, thermodynamic integration by various kinds of Monte Carlo methods have already been developed and used to compute the Bayes factor [13], [17], [38]. Thus, those methods and population annealing can support each other to compute the Bayes factor.

For model selection, we needed to compute the marginal likelihoods of the competing models one-by-one. However, SMC can perform model selection among a number of competing models at one time [15], [18]. One solution to overcome this weak point in population annealing is to use reversible jump MCMC [46] which sampler can jump among parameter subspaces of different dimensions. By jumping among a number of competing models with different parameter dimensions, we can conduct Bayesian model selection by population annealing at one time. This will be a future expansion of population annealing.

Although population annealing still has a room for improvement, population annealing will help us to conduct Bayesian model selection, parameter inference and subsequent simulations with the posterior parameter ensemble for better understanding and prediction of various biological phenomena in system level as shown in this study.

## Supporting Information

Figure S1 **Reproduction and prediction of the large scale observed data.** (A) Simulations with the posterior parameter ensemble of the coherent FFL model in response to the step stimulation of *X*. (B) Simulations with the posterior parameter ensemble of the incoherent FFL model in response to the step stimulation of *X*. (C) Simulations with the posterior parameter ensemble of the coherent FFL model in response to the pulse stimulation of *X*. (D) Simulations with the posterior parameter ensemble of the incoherent FFL model in response to the pulse stimulation of *X*. Blue-colored area is the probability density consists of the simulated trajectories. Red points are the observed data.
(PDF)

Table S1 **Observed data in response to the step stimulation of** ***X*** **.**
(PDF)

Table S2 **Observed data in response to the pulse stimulation of** ***X*** **.**
(PDF)

Text S1 **Supporting algorithms.**
(PDF)

Text S2 **Population annealing in the conventional framework.**
(PDF)

Text S3 **Test with large data.**
(PDF)
