# Supplementary figures and images for: Bayesian Parameter Inference and Model Selection by Population Annealing in Systems Biology
Source: PLoS One. 2014 Aug 4;9(8):e104057. doi: 10.1371/journal.pone.0104057 (PMC4121267; doi:10.1371/journal.pone.0104057)

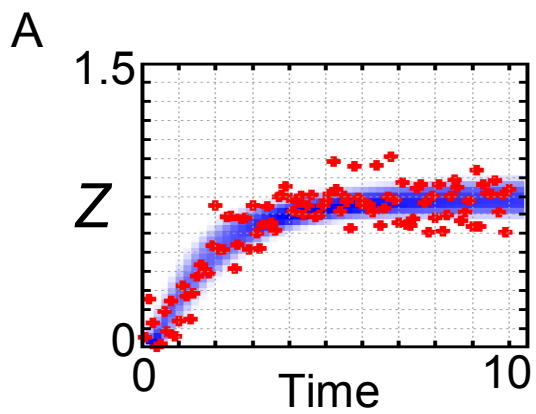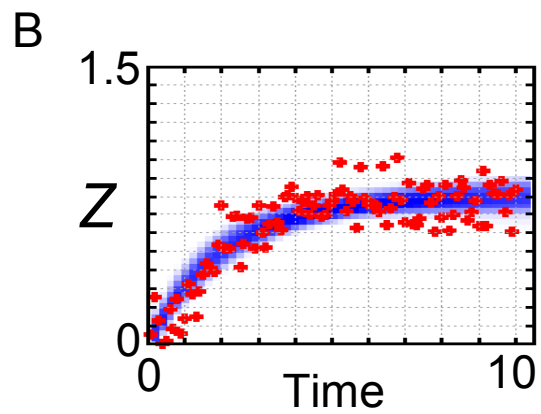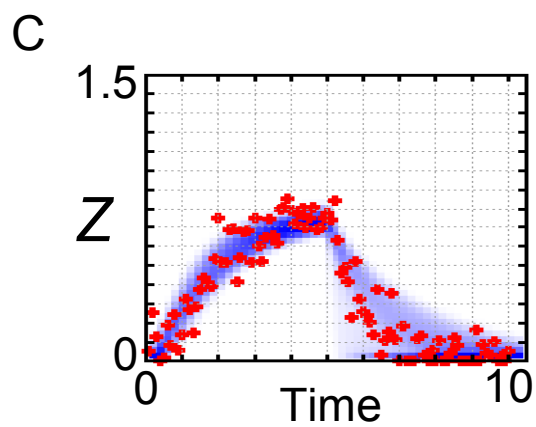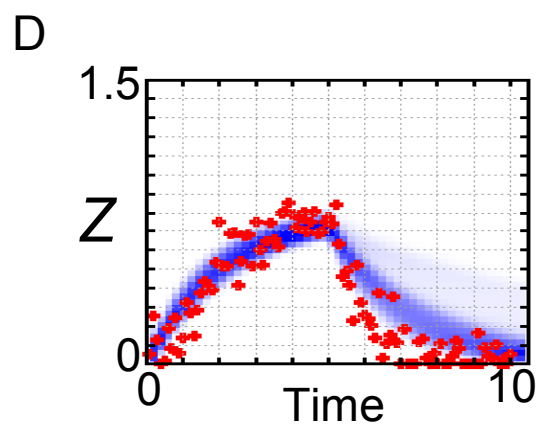

Supplement: Figure S1 — Reproduction and prediction of the large scale observed data. (A) Simulations with the posterior parameter ensemble of the coherent FFL model in response to the step stimulation of X. (B) Simulations with the posterior parameter ensemble of the incoherent FFL model in response to the step stimulation of X. (C) Simulations with the posterior parameter ensemble of the coherent FFL model in response to the pulse stimulation of X. (D) Simulations with the posterior parameter ensemble of the incoherent FFL model in response to the pulse stimulation of X. Blue-colored area is the probability density consists of the simulated trajectories. Red points are the observed data. (PDF) [file pone.0104057.s001.pdf]
