# Supplementary material for: Bayesian Parameter Inference and Model Selection by Population Annealing in Systems Biology
Source: PLoS One. 2014 Aug 4;9(8):e104057. doi: 10.1371/journal.pone.0104057 (PMC4121267; doi:10.1371/journal.pone.0104057)
Supplement: Table S2 — Observed data in response to the pulse stimulation of X . (PDF) [file pone.0104057.s003.pdf]

Table S2. Observed data in response to the pulse stimulation of *X*.

Time	*Z*
1	0.298241
2	0.777959
3	0.780480
4	0.613252
5	0.712871
6	0.367122
7	0.047864
8	0.109689
9	0.000000
10	0.000000
