# Supplementary material for: Bayesian Parameter Inference and Model Selection by Population Annealing in Systems Biology
Source: PLoS One. 2014 Aug 4;9(8):e104057. doi: 10.1371/journal.pone.0104057 (PMC4121267; doi:10.1371/journal.pone.0104057)
Supplement: Text S1 — Supporting algorithms. (PDF) [file pone.0104057.s004.pdf]

Text S1. Supporting algorithms.

1. Markov chain Monte Carlo (MCMC) algorithm

Markov chain Monte Carlo (MCMC) Metropolis-Hastings algorithm proceeds as follows:

M1. Initialize θ_i i = 0.

M2. Propose a candidate value $\theta^* \sim q(\theta|\theta_i)$ where q is a proposal distribution.

M3. Set $\theta_{i+1} = \theta^*$ with following probability α.

$$\alpha = min\left\{1, \frac{f(D_{obs} | \theta^*, M)\pi(\theta^* | M)q(\theta_i | \theta^*)}{f(D_{obs} | \theta_i, M)\pi(\theta_i | M)q(\theta^* | \theta_i)}\right\}$$

Otherwise set $\theta_{i+1} = \theta_i$.

M4. If i < *Repeat number*, increment i = i + 1 and go to M2.

MCMC algorithm is designed as the stationary distribution is consistent with the posterior distribution $\pi(\theta|D_{obs},M)$.

2. ABC-MCMC algorithm

ABC-MCMC algorithm proceeds as follows:

AM1. Initialize θ_i, $D_{sim|\theta i}$ i = 0.

AM2. Propose a candidate value $\theta^* \sim q(\theta|\theta_i)$ where q is a proposal distribution.

AM3. Simulate $D_{sim|\theta^*} \sim f(D_{sim}|\theta^*,M)$.

AM4. Set $\theta_{i+1} = \theta^*$ and $D_{sim|\theta i+1} = D_{sim|\theta^*}$ with following probability α.

$$\alpha = min\left\{1, \frac{f_w\left(D_{obs} \mid D_{sim|\theta^*}, \theta^*, M\right)\pi(\theta^* \mid M)q(\theta_i \mid \theta^*)}{f_w\left(D_{obs} \mid D_{sim|\theta i}, \theta_i, M\right)\pi(\theta_i \mid M)q(\theta^* \mid \theta_i)}\right\}$$

Otherwise set $\theta_{i+1} = \theta_i$ and $D_{sim|\theta i+1} = D_{sim|\theta i}$.

AM5. If i < *Repeat number*, increment i = i + 1 and go to AM2.

ABC-MCMC algorithm is designed as the stationary distribution is consistent with the augmented posterior distribution $\pi_{ABC}(\theta,D_{sim}|D_{obs},M)$.

3. ABC rejection sampler algorithm

ABC rejection sampler algorithm with use of the indicator function as the weighting function ($f_w(D_{obs}|D_{sim},\theta,M) \propto I(d(D_{obs},D_{sim|\theta}) \leqq \varepsilon, M)$) proceeds as follows:

AR1. Sample $\theta^* \sim \pi(\theta|M)$.
AR2. Simulate $D_{sim|\theta^*} \sim f(D_{sim}|\theta^*,M)$.
AR3. If $d(D_{obs},D_{sim|\theta^*}) \leqq \varepsilon$, accept θ^*, otherwise reject.
AR4. Go to AR1.

The samples obtained by ABC rejection sampler follow the augmented posterior distribution $\pi_{ABC}(\theta,D_{sim}|D_{obs},M)$ with setting $f_w(D_{obs}|D_{sim},\theta,M) \propto I(d(D_{obs},D_{sim|\theta}) \leqq \varepsilon, M)$.
