# Supplementary material for: Bayesian Parameter Inference and Model Selection by Population Annealing in Systems Biology
Source: PLoS One. 2014 Aug 4;9(8):e104057. doi: 10.1371/journal.pone.0104057 (PMC4121267; doi:10.1371/journal.pone.0104057)
Supplement: Text S2 — Population annealing in the conventional framework. (PDF) [file pone.0104057.s005.pdf]

Text S2. Population annealing in the conventional framework.

We show intermediate distributions in the conventional framework that a likelihood function can be defined and evaluated. We define the n-th ($0 \leqq n \leqq N$) intermediate distribution as follows

$$f_{IM}^{n}(\theta) \propto \pi(\theta \mid D_{obs}, M)^{\beta n} \pi(\theta \mid M)^{1-\beta n} \propto f(D_{obs} \mid \theta, M)^{\beta n} \pi(\theta \mid M) \quad (0 \leq \beta_n \leq 1)$$

β_n is a fictitious inverse temperature. As β_n increases (temperature decreases, so-called "annealing") from 0 to 1, the intermediate distribution gradually changes from the first intermediate distribution (β_0 = 0) corresponding to the prior distribution, to the last intermediate distribution (β_N = 1) corresponding to the posterior distribution. The concrete schedule of β_n is determined depending on the problem.
