# Supplementary material for: Bayesian Parameter Inference and Model Selection by Population Annealing in Systems Biology
Source: PLoS One. 2014 Aug 4;9(8):e104057. doi: 10.1371/journal.pone.0104057 (PMC4121267; doi:10.1371/journal.pone.0104057)
Supplement: Text S3 — Test with large data. (PDF) [file pone.0104057.s006.pdf]

Text S3. Test with large data.

To investigate the influence of the data scale, we conducted the test with the observed data consists of 100 time points of *Z* in the FFL models. We artificially generated the observed data consists of 100 time points of *Z* (red points in Figure S1.A, B) from the coherent FFL model (Figure 1.A) with the same answer values of parameters and Gaussian noise shown in the main text. The data was generated in response to the step stimulation of *X* (Figure 3.A) and used for all the computations in this Text S3. Here, all the computations by population annealing employed the annealing schedule ($\varepsilon_0, \varepsilon_1, \varepsilon_2, \varepsilon_3, \varepsilon_4, \varepsilon_5$) = ($\infty$, 10, 5, 2.5, 2, 1.5).

Firstly, we conducted parameter inference and ran the simulations with the posterior parameter ensemble in response to the step stimulation of *X*. As shown in Figure S1.A, the simulations with the posterior parameter ensemble of the coherent FFL model could capture the observed data used for parameter inference. In addition, the simulations of the incoherent FFL model could also capture the observed data as shown in Figure S1.B. These results are similar to those with the small data consists of 10 time points of *Z* (Figure 3, 6).

To test predictability, we ran the simulations with the posterior parameter ensemble in response to the pulse stimulation of *X*. The observed data consists of 100 time points of *Z* (red points in Figure S1.C, D) in response to the pulse stimulation of *X* was newly generated adding the same Gaussian noise in Figure S1.A and B. The simulations with the posterior parameter ensemble of the coherent FFL model could capture and predict the observed data which was not used for parameter inference (Figure S1.C). However, the simulations of the incoherent FFL model could not entirely capture and predict the data compared with the coherent FFL model. Concretely, the trajectories after time = 5 could not capture the observed data well in Figure S1.D. These results are also similar to those with the small data consists of 10 time points of *Z* (Figure 4, 7).

Lastly, we conducted Bayesian model selection. The mean and the standard deviation of the Bayes factor B^{ABC}_{CI} computed by population annealing was 2.418 ± 0.076. In addition, the mean and the standard deviation of the Bayes factor computed by ABC rejection sampler was 2.464 ± 0.094. Thus, the coherent FFL model was selected as

well as the result shown in Table 1.

Taken together, the results with the large data were similar to those with the small data shown in the main text.
